# Traumatized German soldiers with moral injury – value-based cognitive-behavioral group therapy to treat war-related shame

**DOI:** 10.3389/fpsyt.2023.1173466

**Published:** 2023-07-18

**Authors:** Caroline Diekmann, Leonie Issels, Christina Alliger-Horn, Heinrich Rau, Christian Fischer, Thomas Thiel, Gerd Willmund, Peter Zimmermann

**Affiliations:** ^1^Department of Psychiatry, Psychotherapy and Psychotraumatology, Military Hospital Berlin, Berlin, Germany; ^2^Charité - Berlin University of Medicine, Humboldt University of Berlin, Berlin, Germany; ^3^Military Chaplaincy Central Office, Berlin, Germany

**Keywords:** moral injury, shame, values, soldiers, military, posttraumatic, group therapy, PTSD

## Abstract

**Introduction:**

During deployment, soldiers are confronted with potentially morally injurious events. In many cases, these events violate their personal values and belief systems, resulting in feelings of anger, alienation, guilt, and shame. The psychological distress caused by such transgressions is defined as moral injury. It remains unclear to date, which therapeutic interventions are most appropriate for addressing this specific psychological condition. This study examines the effectiveness of value-based cognitive-behavioral group therapy combining elements of cognitive-behavioral therapy, acceptance and commitment therapy, spiritual care, and adaptive disclosure therapy.

**Materials and methods:**

This controlled study uses the Compass of Shame Scale to assess symptom severity among participants both before and after a three-week inpatient group therapy regimen for moral injury. An intervention group (*n* = 45) was compared to a waiting-list control group (*n* = 40). A one-way between subjects ANOVA was conducted to determine the differences between the two measurement points in the intervention group compared to the control group. A positive ethics vote from the Humboldt University Berlin (Charité) was available (No.EA1/092/15).

**Results:**

A significant difference was found on the shame-associated maladaptive strategies subscales of attack self (*F* (1, 83) = 5.942, *p* = 0.017, Cohen’s *f* = 0,27), withdrawal (*F* (1, 83) = 8.263, *p* = 0.005, Cohen’s *f* = 0,32), and attack others (*F* (1, 83) = 10.552, *p* = 0.002, Cohen’s f = 0,36) of the Compass of Shame Scale between the intervention group and the control group at the *p* < 0.05 level in the pre- and post-treatment (t1-t2) comparison.

**Conclusion:**

This study suggests that the special therapeutic focus in cognitive-behavioral group therapy can alter shame-based maladaptive coping behaviors in response to war-related moral injury. This study provides further evidence that therapeutic approaches – through fostering a reconciliatory, compassionate, and forgiving approach toward oneself and others – target the underlying mechanisms of moral injury. Therefore, value-based cognitive-behavioral interventions should be considered as a standard element of trauma care in a military setting. Future studies should further examine such interventions in randomized control trials. It would also be particularly valuable for future studies to include a follow-up time point.

## 1. Introduction

In unusually high-stress situations during deployment, soldiers are at risk of encountering events that transgress their deeply embedded values and belief systems. The experience of failing to prevent immoral behavior by others or having behaved profoundly immorally oneself may lead to a subjectively unresolvable conflict between basic internal beliefs, schemas, and lived experience (1–3). Those moral violations are considered potentially morally injurious events (PMIEs), as are feelings of being deceived by a military leader or institution (4). The severe psychological, social and spiritual repercussion caused by those experiences are known as moral injury (MI) (5–7).

The inability to forgive oneself and others seems to be central to the concept of MI (8), leading to a shift in an individual’s understanding of values and norms along with disturbances in self-referential processing (9–11). Kopacz discusses MI as a “primary psychological injury” (12) that is not fully captured by the conventional diagnostic criteria of post-traumatic stress disorder (PTSD) but can instead be understood as a dimensional construct. The author describes a crucial correlation between feelings mediated by deployment-related MI and PTSD symptoms such as overlaps in intrusive re-experiencing (intrusions), emotional flattening and avoidance behavior. Other studies have indicated that Moral Injury (MI) is a concept that is functionally distinct from PTSD, despite the frequent overlap of symptoms. Differences can be observed in terms of etiology and resulting symptoms. The symptom profiles of posttraumatic stress disorder arising from life-threatening and fear-based trauma are characterized by increased vigilance, flashbacks, memory loss, nightmares, and insomnia, whereas the complex of moral injury based on betrayal and perpetration is characterized by high scores for shame, guilt, remorse, disappointment, and feelings of betrayal (7, 13).

In particular, shame is considered a central factor in the highly destructive and self-injurious behavior of traumatized soldiers with a history of PMIE, manifesting in a dysfunctional focus on the immoral self, an avoidance of intimacy and profound self-criticism (14, 15). Similarly, emotional responses elicited by shame appear to underpin a destructive attitude toward the external world, including loss of trust in others, skepticism of authority, loss of former religious beliefs, and loss of faith in a just world (16). Unsurprisingly, feelings of shame, among other conditions, have been identified as key predictors of depression and suicidal behavior in soldiers (17). Other studies have demonstrated that MI in German soldiers is associated with an increased risk of developing PTSD and susceptibility to depression, anxiety, and addictive behavior (11).

In contrast to shame, guilt, although a similarly painful emotion, is described as an adaptive moral emotion resulting in greater empathy for others. Guilt ultimately motivates the individual to choose moral behavior. Thus, unlike shame, guilt exhibits an inhibitory influence on antisocial and risky behavior (18).

Several studies have found prolonged exposure and cognitive processing therapy (CPT) to be acceptable, feasible, and effective treatments for PTSD symptoms (19). There is conjecture that these standard treatments, although originally conceptualized to treat fear-based PTSD, would be equally well suited to treat MI-related symptoms. Like it facilitates the alleviation of PTSD symptoms by challenging dysfunctional cognitions (i.e., stuck points), CPT could also help patients to contextualize their guilt, accept arising emotions and challenge unrealistic cognitions by recognizing and incorporating potentially mitigating external constraints and internal influences, that contributed to the occurrence of a PMIE. Moral recovery may be achieved through prolonged exposure and CPT by accessing avoidance behavior present both in PTSD and MI, being motivated either by fear or shame. These assumptions are congruent with the qualitative description of two successful treatments of veterans with PTSD and PMIE exposure (20).

Yet, several authors have hypothesized that the standard trauma-focused therapies based on a predominantly fear-conditioning and cognitive reappraisal paradigm may be insufficient in treating the difficult and complex psychological responses of MI. For example, a qualitative study revealed that, for individuals with MI, standard interventions have a limited impact on MI-related symptoms, and benefits were not maintained on follow-up (21). Concerns have arisen, that morally injurious outcomes resulting from committing or failing to prevent an act of moral perpetration might remain unchanged by habituation processes and persist even with reappraisal. In response, an increasing number of recently developed interventions seek to directly address specific MI-related symptoms of shame, guilt, remorse, disillusionment, a sense of betrayal and loss of faith. Along with that, there is growing evidence for interventions that aim to facilitate self-forgiveness or forgiveness of others, which could pose a valuable alternative to existing therapy or could usefully complement standard cognitive-behavioral therapy (CBT) (4, 22, 23).

Although several trauma-focused interventions have gained solid empirical support, it remains unclear which interventions are particularly suited to the specific characteristics of MI and which elements are critical determinants of their effectiveness (24). Studies on acceptance and commitment therapy (ACT) have indicated that the symptoms of PTSD and depression could be effectively treated by such interventions, leading to the improvement in the overall well-being of individuals who have been exposed to a PMIE. However, ACT intervention does not accurately target the construct of shame; thus, symptoms of shame were not significantly reduced. At follow-up, the benefits of MI-related outcomes were not sustained. Notably, life satisfaction decreased from the baseline one-month post-intervention (25). Several small studies also support the effectiveness of the Impact of Killing intervention as an adjunctive therapy for MI (26). This approach specifically focuses on self-forgiveness, addressing the symptoms associated with killing in war, namely guilt, shame, and self-sabotaging behaviors (27). Adaptive Disclosure therapy (AD), as a novel approach, similarly aims to encourage self-blame processing, combining various interventions – such as emotion-focused imaginal exposure, cognitive restructuring and loving kindness meditation – in an one-on-one therapeutic setting (9). Indeed, AD has proven non-inferiority to standard Cognitive Processing Therapy in a small randomized trial among service-members with a history of PMIES (28).

Further pilot studies have supported the usefulness of mindfulness-based therapies as adjuncts to standard PTSD treatments (29, 30). Additionally, combined mental health practitioner and chaplaincy interventions, which provide spiritually integrated group counseling to reduce trauma-related symptoms and promote psychospiritual development, have demonstrated clinically and statistically significant improvements in spiritual distress and PTSD symptoms (31, 32). However, since these studies largely lack long-term follow-up data, the sustainability of the interventions’ effects remains unknown.

A newly developed cognitive-behavioral group therapy combining elements of ACT, CBT, spiritual care, and AD therapy was applied in a study conducted at the Bundeswehrkrankenhaus Berlin. The therapist-led intervention integrated cognitive-behavioral work around values and the impact of MI on daily life and emotions as well as the use of compassionate imagery. Conceptualization of this approach was based on clinical experience that a significant number of veterans with MI explicitly address values and norms, including their transformation and upheaval. The specific thematization of value orientations integrating therapeutic aspects of ACT was intended to provide a stabilizing framework for the confrontation with the feelings of shame and guilt following exposure to PMIES. The approach posits that identifying personal values and life goals provides a broader context for the uncertainty and suffering associated with traumatic experiences, enabling the initiation of moral repair through the practice of forgiveness, compassion, and reconciliation with the involvement of spiritual care. The chosen group setting allowed for the benefit of an emerging feeling of togetherness in the group (group cohesion), which can be used therapeutically. The topics discussed served to make participants aware of or reinforce feelings of shame, which have usually already sustained a prolonged process of social isolation. In an appreciative and supportive group setting, a climate of acceptance and thus a corrective communal experience can be created. Likewise, treatment by an interdisciplinary team can promote a stimulating and holistic psychospiritual approach to MI. The treatment described, was recently manualized (33). To date, this controlled study of soldiers with deployment-related disorders was the first outside of the United States of America to evaluate a combined psychotherapeutic approach using shame as an MI-specific outcome variable.

Following a model developed by Nathanson (34), the four poles of the compass of shame characterize different maladaptive strategies in the attempt to avoid, deny, reduce, or amplify shame without addressing its source. The central shame-related strategies frequently encountered in the everyday clinical treatment of soldiers are distinguished as attack self (e.g., self-reproach, masochism), attack others (e.g., verbal and physical aggression toward others), avoidance (e.g., self-isolation, disengagement from the community of family and friends), and withdrawal (e.g., denial, abusing drugs or alcohol, distraction through thrill-seeking). This study is based on the hypothesis that the chosen group therapy will lead to a significant reduction in symptoms related to MI and shame, including withdrawal, attack others, attack self, and avoidance strategies. The use of the four shame coping styles described by Nathanson was examined both before and after the three-week inpatient group therapy regimen for moral injury through the Compass of Shame Scale (CoSS), which has already proven to be a helpful tool for viewing maladaptive strategies for coping with shame (35, 36). Thus, this study contributes to enhancing understanding of the role of shame in processing traumatic experiences.

## 2. Method

### 2.1. Study population

Forty-five soldiers [43 males and two females, with an average age of 39.67 years (SD ± 7.48 years)] with deployment-related chronic PTSD participated in three-week inpatient, semi-standardized group therapy. These participants were compared to a control group of 40 PTSD patients [37 males and three females, with an average age of 40.97 years (SD ± 7.39 years)] who did not undergo treatment. The military engagements in which the soldiers were deployed involved the International Security Assistance Force (ISAF) and Resolute Support (RS) in Afghanistan, the Kosovo Force (KFOR) and the Stabilization Force (SFOR) in Bosnia and Herzegovina, the Operation Active Fence (ATUR) in Turkey, the Mission Counter Daesh and Capacity Building in Jordan and Iraq, the United Nations Interim Force in Lebanon (UNIFIL), the United Nations Multidimensional Integrated Stabilization Mission in Mali (MINUSMA) and the United Nations Advance Mission in Cambodia (UNAMIC), and the European Union Training Mission (EUTM) and the European Union Naval Force (EU NAVFOR) in Somalia. All patients reported specific experiences with the character of MI at initial diagnosis, listing of multiple incidents was permitted, namely killing of combat opponents (*n* = 15), serious injury/death of comrades (*n* = 25), suicide of comrades with feelings of guilt for the inability to prevent his/her death (*n* = 3), witnessing violence and atrocities toward non-combatants [especially against women and children (e.g., rape or stoning)] (*n* = 15), finding mutilated bodies (including children after organ removal or severe abuse) (*n* = 10), failure to provide aid or rescue measures for wounded or perpetrated persons (e.g., for civilians or allied forces) (*n* = 6), refusal to help locals in need (e.g., in medical care against the background of special security regulations or orders) (*n* = 2), witnessing brutal/violent consequences for locals after military intelligence acquisition (*n* = 4), Interrogation of children (*n* = 1), targeted killing of a child with an explosive belt and subsequent removal of body parts (*n* = 1), killing of a child (*n* = 1), futility of withdrawing from Iraq (*n* = 1), betrayal or insult by comrades and superiors (*n* = 9), and facing hostility from civilians (e.g., assault by children begging for water) (*n* = 6). KFOR veterans further reported seeing mass graves with exhumation of decomposed bodies (including women and children) (*n* = 2) and finding a burning mass grave (*n* = 1).

### 2.2. Research procedures

All patients gave informed consent for an examination and voluntarily participated in the group therapy. A positive ethics vote from the Humboldt University of Berlin (Charité) was available (No. EA1/092/15). Participants in the intervention group were required to meet the following inclusion criteria: voluntary participation in group therapy, a PTSD diagnosis according to ICD-10/DSM-V by a psychiatric specialist, and the presence of moral distress related to war trauma. Individuals with acute suicidality, psychosis, or unstable addictions were excluded from participation. Patient assignments were conducted in the clinic’s psychiatric ward, with group sizes ranging from three to eight (average of six) participants.

### 2.3. Measures

Forty-five soldiers [43 males and two females, with an average age of 39.67 years (SD ± 7.48 years)] with deployment-related chronic PTSD participated in three-week inpatient, semi-standardized group therapy. These participants were compared to a control group of 40 PTSD patients [37 males and three females, with an average age of 40.97 years (SD ± 7.39 years)] who did not undergo treatment. Patients in the intervention group were examined at two measurement time points: before the start of group therapy (t1) and immediately following the end of group therapy (t2). No treatment discontinuations occurred. The control group patients were examined at outpatient presentation (t1) and 3–4 weeks later (t2) without having received psychotherapy.

MI symptom severity was assessed using the CoSS, which has four subscales with acceptable to good internal consistencies (Cronbach’s alpha: withdrawal = 0.89, attack other = 0.85, attack self = 0.91, avoidance = 0.74) (37). It describes different dysfunctional coping strategies regarding experienced shame according to Nathanson’s model (34): The avoidance strategy is used to unconsciously suppress or deny the feeling of shame, or to make mostly unconscious efforts to distract oneself from the feeling of shame. The perception of shame or the recognition of the triggering event is mostly limited. The attack other strategy is motivated by an externalization of shame as well as an outward directed anger, which is exercised in an action-orientated manner by verbal or physical attacks on subjects or objects. With this strategy the feeling of shame and also the triggering event as well as the misconduct of another person may be consciously recognized. For the withdrawal strategy, the affected person tries to disengage or hide from social situation and the feeling of shame is not necessarily identified by the individual. However, thoughts such as “I am worthless” are recognized as valid. On a cognitive level, one’s own actions or character traits are perceived as wrong or bad, and the person feels worthless in the presence of others. The attack self strategy also involves a negative experience together with an internalized feeling of worthlessness. The anger is directed inward, and emotions such as contempt or disgust also occur. In this process, the person is aware of actions or characteristics that are judged as bad. Similar to withdrawal, negative feelings and cognitions can be acknowledged without being explicitly recognized as shame. The motivation is to gain control over the shame, with the ultimate goal of being accepted by others. The action tendency is to criticize oneself, to prevent recurrence of the shameful situation by changing, to adjusting, expressing respect for others, or making self-deprecating remarks. Through the CoSS 12 daily life situations or self-related appraisals are presented with four possible coping strategies. The strategies are rated according to the degree to which they are likely to be chosen. An illustrative statement such as “when I think I have disappointed other people” is to be rated according to the frequency of the possible subsequent reactions in everyday life such as “I get mad at them for expecting so much from me.” (attack others), “I cover my feelings with a joke” (avoidance), “I get down on myself” (attack self) and “I remove myself from the situation.” (withdrawal) (36).

The Federal Language Office translated and re-translated the CoSS for the purpose of this study. Validation of the German version is in preparation.

### 2.4. Therapeutic intervention

Patients in the intervention group were treated using value-based cognitive-behavioral semi-standardized group therapy divided between one and three 90-min daily sessions over 3 weeks, resulting in a total of 20 sessions. Effective preparation, follow-up, and the establishment of a trusting atmosphere made a decisive contribution to the ability to cope with the challenging topic and the high therapeutic density during the second week of therapy. To this end, the first week focused on building group cohesion to allow for the exploration of values and moral transgressions in a supportive atmosphere during the therapy sessions of the second week. The third week provided a reflective phase to consolidate the knowledge gained throughout the treatment. The therapy sessions were conducted under the supervision of a psychiatrist and a licensed psychotherapist (both qualified in group psychotherapy and special psychotraumatology under the German Trauma Association, DeGPT). The contents of the individual therapy sessions are described below. Table 1 provides an overview of the various thematic blocks, guiding questions, and accompanying exercises of the therapeutic manual upon which this study is based.

**Table 1:** Overview of the treatment components and exercises covered at each session of a value-based cognitive-behavioral group therapy to treat war-related shame.

Sessions	Topics	Exemplarly guiding questions	Optional exercises
1–3	Psychoeducation on the topics of war trauma and affect regulation, Identification of everyday destructive behaviors toward others (e.g., aggressive social interaction) and oneself (e.g., excessive exercise, unhealthy eating habits)	How are one’s own needs and resources dealt with? Are self-forgiveness and self-consolation possible in stressful conflict situations? How are conflicts managed and frustration coped with?	Compassion-focused imagery guided breathing exercises Trauma-releasing exercises (TRE)
4–5	Improvement of social competencies and interactions, identification of personal resources	What do you genuinely care about? What do you want your life to stand for?	Meditation Mindful walking
6–7	The importance of value orientations in daily life and the advantages and disadvantages deriving therefrom	What are values and why are value orientations important for individuals? What consequences can rigid, uncompromising values have?	Group discussion
8–9	Contextualisation of values and belief systems and deployment-related changes	How did participation in the deployment affect personal value orientations? What are the advantages and disadvantages associated with changed values and belief systems in the areas of perception of service and private life?	Imaginative value affirmation (imaginative description of a future characteristic scene representing the desirable new values (“Me in 10 years”))
10–13	Moral Injury (MI) resulting from the moral misconduct of others	Why did the perpetrator commit his actions? *Therapists may adopt a judgmental position in order to be authentically perceptible for the participants (“…that was a terrible offence…”), if necessary, this should also be put into perspective: (“Was the perpetrator perhaps overtaxed himself?”).* What consequences does anger have for the relationships with their close relatives (e.g., bitterness, relationship conflicts, separation…)? Is anger still “attractive” when these changes are taken into account?	e.g.,Thought-stopping techniques, compassionate imagination and developing potential healing cognitions
14–17	MI resulting from one’s own moral misconduct	Why did the person act in that way in that situation? What consequences did the behavior lead to for themselves and for others? What personal value orientations and moral standards might be in conflict with such behaviors? Accordingly, what thoughts, feelings or symptoms might have developed?	Inner judge or trainer, imaginary dialogue with a benevolent moral authority practice of forgiveness using compassionate imagery
18–20	Integration of solutions to moral conflicts into daily life	How does shame affect self-esteem? What are the consequences of shame for dealing with oneself? Can shame also have advantages? What does the fact that feelings of guilt and shame have arisen at all in the face of the traumatic situation say about those affected? How can meaningful and supportive personal social relationships be resumed?	Group discussions and role play

#### Sessions 1–3

To achieve a common level of knowledge among participants during the first sessions, psychoeducation on the topics of war trauma, MI, PTSD, and affect regulation was provided. Additionally, instruction was given on self-observing maladaptive behavioral strategies. The focus was on recognizing everyday destructive behaviors toward others (e.g., aggressive social interaction) and oneself (e.g., excessive exercise, unhealthy eating habits) as maladaptive behavioral strategies. Gilbert’s affect regulation model (38) was discussed, and compassionate imaginary procedures were practiced.

#### Sessions 4–5

These sessions aimed to improve and enhance social competencies and interactions, personal resources, mindfulness, and problem-solving strategies.

#### Sessions 1–5

These sessions aimed to build group cohesion among the participants to facilitate engagement with the more challenging topics addressed in sessions 6–20.

#### Sessions 6–17

The patients at this stage motivated to understand deployment-related changes in their values and belief systems holistically. A closed setting (e.g., hotel, monastery) was chosen to ensure a pleasant ambiance and appropriate familiarity, provide security, and signify appreciation, was chosen to address the participants` lowered self-esteem. These sessions were conducted by an interdisciplinary therapeutic team (psychiatry, psychology, pastoral care, and nursing), enabling the professionals to consider the discussed topics from various perspectives.

#### Sessions 6–7

During these sessions, participants were asked to share the importance of their value orientations in daily life and the perceived advantages and disadvantages. For example, participants considered the guiding question is, “What are the advantages and disadvantages of a value-based or a materialistic way of life?”

#### Sessions 8–9

These sessions contextualized value orientations regarding deployment experiences. The guiding questions included the following: “How did participation in the deployment affect personal value orientations?” “In which areas did they change?” and “What are the advantages and disadvantages associated with this change in the areas of perception of service and private life?”

#### Sessions 10–13

These sessions focused on MI resulting from the moral misconduct of others. Participants offered examples of mission-related moral conflict or selected examples from an existing list. These examples led to exploring and discussing participants’ emotional responses, such as disappointment, embitterment, or anger, and their potentially destructive effects on the participants’ lives. Subsequently, ways of practicing forgiveness (e.g., compassionate imagination and developing potential healing cognitions) following moral violation were addressed.

#### Sessions 14–17

These sessions centered on the participants’ perceptions of their moral misconduct. The participants’ discussions were guided toward the possible consequences of guilt and shame for one’s inner experience and social contacts and concluded with an elaboration on healing cognitions (e.g., inner judge or trainer, imaginary dialogue with a benevolent moral authority, and forgiveness using compassionate imagery).

#### Sessions 18–20

These sessions aimed to use psychoeducation, group discussion, and role-play exercises to integrate solutions to moral conflicts into daily life, for example, managing aggression toward others or oneself in private and official contexts (33, 39).

### 2.5. Statistics

The data in this study were analyzed using SPSS version 25.0 for macOS [IBM SPSS Statistics for Macintosh, Version 25.0. Armonk, NY: IBM Corp (2017)].

A one-way between-subjects analysis of variance (ANOVA) was conducted on the delta scores, representing the differences in symptom severity between the post-intervention (t2) and pre-intervention (t1) measurements for each subscale within each group. The delta scores in the intervention group were derived by subtracting the symptom severity at the post-treatment measurement (t2) from the symptom severity at the first outpatient presentation (t1). Similarly, in the control group, the delta scores were obtained by comparing the symptom severity at the first outpatient presentation (t1) with the symptom severity measured 3–4 weeks later without receiving psychotherapy (t2). This design ensured that the delta scores captured the change in symptom severity over time within each group, allowing for a direct comparison between the intervention and the control group.

The primary objective of the ANOVA on the delta scores was to rigorously examine the observed differences in symptom severity change between the two groups, thereby providing a robust statistical assessment of the intervention’s efficacy. Furthermore, effect sizes were quantified using Cohen’s f, allowing for a precise estimation of the magnitude and practical significance of these observed differences. This analytical framework furnished a comprehensive evaluation of the impact of the intervention on the alterations in symptom severity.

## 3. Results

The analysis of age, gender, and symptom severity revealed no significant differences in baseline data between the intervention and control groups (data not shown). Table 2 displays the results of the ANOVA test, revealing significant differences in the change in symptom severity between the intervention and control groups for three out of four subscales of the CoSS (attack self, withdrawal, attack others), indicating that the intervention had a positive effect on reducing maladaptive shame-associated strategies compared to the control group. The effect sizes (Cohen’s f) observed in these results were small.

**Table 2:** One-way ANOVA demonstrating significant differences in change in symptom severity between intervention and control group for three out of four subscales of the CoSS.

	Mean (SD) Intervention group	Mean (SD) Control group	*F*	Sig (p)	η^2^ (partial)	Effect size (Cohen’s f)
Delta_AV (t1-t2)	1,53 (SD = 7,689)	−1,47 (SD = 6,583)	3,701	0,058	0,043	0,21
Delta_AS (t1-t2)	2,91 (SD = 8,570)	−1,85 (SD = 9,437)	5,942	0,017	0,067	0,27
Delta_WD (t1-t2)	3,87 (SD = 7,269)	−0,93 (SD = 8,100)	8,263	0,005	0,091	0,32
Delta_AO (t1-t2)	3,62 (SD = 5,553)	−0,75 (SD = 6,845)	10,552	0,002	0,113	0,36

SD, standard deviation; F, *F*-value; *p*-value, probability value; η2 (partial), partial eta-squared; t1 = first outpatient presentation, t2 = post-treatment (intervention group) or 3–4 weeks later without having received psychotherapy (control group), Effect size = ${\mathrm{Cohen}}^{\prime }s\;f=\surd \left(\;\eta^{2}/\left(\;1-\eta^{2}\right)\right)$ . Compass of Shame subscales: AV, avoidance, AS, attack self, WD, withdrawal, AO, attack others. Negative delta values describe an increase in the severity of shame-related symptoms.

## 4. Discussion

This study has focused on the specifics of MI and its potential for effective therapy within a cognitive-behavioral treatment program for German soldiers. At this point in time, comparable military studies outside of the Unites States of America with shame as the primary outcome variable are not available in the literature. The results of this study suggest that this therapeutic focus can alter shame-based maladaptive coping strategies in response to war-related trauma. According to the results, the extent of shame-related aggressive and destructive coping behaviors toward the self and others significantly decreased between the two measurement time points. Specifically, the intervention group exhibited significant reductions in the attack self, attack others, and withdrawal subscales compared to the waiting-list control group.

Gray et al. (9) among others, describe MI as a syndrome of shame and associated self-destruction. Failing to prevent immoral behavior in war situations, transgressing one’s moral standards, or being a victim of others’ misconduct frequently leads to maladaptive processing and MI, which is associated with feelings of shame and anger.

Examining personal values and moral beliefs and their impact on mental health following deployment has yielded clear indication of the importance of these topics in the context of traumatized soldiers (10, 11, 14). However, as this study’s approach combines elements of ACT, CBT, spiritual care, and AD therapy, the extent to which any of these approaches alone contributes to therapeutic effects remains unclear. Furthermore, comparability across different studies remains limited due to the use of different outcome measures.

As previously stated, Litz et al. (29) and Steenkamp et al. (23) have studied AD with positive results. Whether “dialogue with a compassionate and forgiving moral authority” explains significant elements of the symptom reduction in this study must be further explored. Alliger-Horn et al. (16, 39) has further demonstrated the effectiveness of developing a compassionate imaginary about one’s traumatized self in changing trauma-related feelings of guilt and shame. These findings also align with patients’ statements in qualitative interviews on impact of killing interventions; patients identified self-forgiveness as fundamental for the beneficial therapeutic effects (15, 26).

In contrast, when examining an intervention with self-forgiveness as an adjunctive therapy to CPT group therapy, Snider et al. (40) observed no significant differences between a control group and an intervention group in experience of shame or MI. This finding suggests that additional components are required to treat shame and MI successfully. The combined ACT and CBT elements of this study could play this crucial role (23, 26).

The group’s mindfulness-based and spiritual approach in this research could have contributed to its therapeutic impact. In previous research, combined interventions by psychotherapists and chaplains predominantly focused on spiritual development and reducing spiritual distress resulted in clinically and statistically significant improvements in PTSD symptoms as well as benefits in post-traumatic growth and self-competence (31, 32, 41). Broader insights into the determining factors of the therapeutic efficacy of the intervention applied may be obtained in future qualitative studies through examining patients’ perspective.

## 5. Study limitations

This study has several limitations, including the small sample size and the lack of randomization. Since the treatment program includes therapeutic elements in addition to the core themes of MI, it cannot be proved that the therapeutic effect can contribute to moral topics alone. In addition, the sample was limited to soldiers who had experienced deployment-related traumatic events. Therefore, the study findings have limited applicability to other trauma groups. Furthermore, the study does not yet address long-term effects.

## 6. Conclusion

Despite several limitations, the study results suggest that a CBT approach focusing on value changes in patients’ moral constructs and developing a reconciliatory, compassionate, and forgiving attitude toward the self and others could address the underlying mechanisms of MI and should, therefore, be at the core of value-based cognitive-behavioral interventions. Since many soldiers experience a wide range of deployment-related psychological conditions, further studies should combine this study’s approach with examination of other trauma-related psychotherapy approaches. In particular, the deployment of value-based primary and secondary prevention before and after deployment deserves further attention. In workplaces with a predictable risk of exposure to PMIEs (e.g., emergency response, humanitarian aid, journalism), skills training focusing on moral issues should be implemented to support moral-incident preparedness.

## Data availability statement

The raw data supporting the conclusions of this article will be made available by the authors, without undue reservation.

## Ethics statement

The studies involving human participants were reviewed and approved by Humboldt University of Berlin (Charité). The patients/participants provided their written informed consent to participate in this study.

## Author contributions

CD, LI, CA-H, GW, HR, and PZ contributed to conception and design of the study. CF and CD contributed to the conception of the manual. CD performed the statistical analysis and wrote the first draft of the manuscript. All authors contributed to the article and approved the submitted version.

## Conflict of interest

The authors declare that the research was conducted in the absence of any commercial or financial relationships that could be construed as a potential conflict of interest.

## Publisher’s note

All claims expressed in this article are solely those of the authors and do not necessarily represent those of their affiliated organizations, or those of the publisher, the editors and the reviewers. Any product that may be evaluated in this article, or claim that may be made by its manufacturer, is not guaranteed or endorsed by the publisher.
